# Integrative Medicine for Cancer-Related Pain: A Narrative Review

**DOI:** 10.3390/healthcare12030403

**Published:** 2024-02-04

**Authors:** Noah Samuels, Eran Ben-Arye

**Affiliations:** 1 Center for Integrative Complementary Medicine, Shaare Zedek Medical Center, Faculty of Medicine, Hebrew University of Jerusalem, Jerusalem 9103102, Israel; 2 Integrative Oncology Program, The Oncology Service, Lin Carmel, and Zebulun Medical Centers, Clalit Health Services, Haifa 3535152, Israel; eranben@netvision.net.il; 3 Department of Family Medicine, Faculty of Medicine, Technion-Israel Institute of Technology, Haifa 3200003, Israel

**Keywords:** cancer-related pain, complementary integrative medicine, acupuncture, touch therapies, mind-body-medicine, herbal supplements, pragmatic research

## Abstract

Cancer-related pain (C-RP) is a prevalent and debilitating concern among patients with cancer, with conventional treatments limited in their ability to provide adequate relief, and by the adverse effects associated with their use. Complementary and integrative medicine (CIM) modalities have been shown to be potentially effective and safe for the treatment of pain and related symptoms, when used in conjunction with conventional medications and under medical supervision. An increasing number of oncology centers provide CIM within their conventional supportive and palliative care service, in an “Integrative Oncology” (IO) setting. A large body of clinical research, including systematic reviews and guidelines such as those published in 2022 by the Society for Integrative Oncology (SIO), in collaboration with the American Society for Clinical Oncology (ASCO), support the use of some CIM modalities for C-RP and related concerns. These include acupuncture for general and peri-operative/procedural pain, as well as aromatase inhibitor-associated arthralgia (AIA); reflexology or acupressure for pain during systemic therapy for cancer; hypnosis for procedural pain or pain due to diagnostic workup; and massage for pain experienced by patients during palliative and hospice care. Further research is needed, within both randomized control trials and pragmatic non-controlled studies which are more reflective of the real-life IO setting. This review summarizes the evidence supporting the use of CIM for C-RP; the analgesic mechanism of the modalities presented; and the challenges facing IO researchers, as well as the implementation of the 2022 SIO-ASCO guideline recommendations.

## 1. Introduction

Cancer-related pain (C-RP) is one of the most common and debilitating concerns reported by oncology patients [1], and is invariably associated with severely impaired quality of life (QoL) and function [2,3,4]. The pathogenesis of C-RP is complex and multifactorial, reflecting processes such as local and metastatic tumor invasion of adjacent organs; peri-operative pain; pain experienced during palliative and end-of-life care; and oncology treatment-related symptoms such as chemotherapy-induced peripheral neuropathy (CIPN) and aromatase inhibitor-induced arthralgia (AIA) [5,6]. The symptoms of C-RP can be severe, often persisting throughout survivorship and end-of-life care. While conventional medicine can offer patients a wide range of treatments for C-RP, including non-opioid and opioid analgesic drugs, these do not always provide adequate pain relief, and may be accompanied by significant adverse effects which limit their use [7]. In many cases, C-RP is associated with reduced adherence by patients to the recommended oncology treatment regimen, requiring dose reductions or a decision to switch to second or third-line drugs, with implications regarding outcomes such as survival and prevention of disease recurrence [8,9]. It is therefore important to examine other treatment options for the relief of C-RP, to be used in addition to available conventional treatments for this indication which are, at the same time, medically appropriate and consistent with the goals of care [10].

Complementary and integrative medicine (CIM) is a term used to describe a shared work environment in which complementary medicine practices are provided within the conventional medicine setting [11]. Many of today’s leading oncology centers in the U.S. and across the globe are including CIM as part of their supportive and palliative oncology services, in an “Integrative Oncology” (IO) setting [12,13]. The CIM treatments provided in these IO services are patient-centered and evidence-informed, with the stated goal of optimizing the patient’s health, quality of life, and clinical outcomes throughout the continuum of cancer care [14]. The most common CIM practices being provided in IO settings include acupuncture; massage and touch (e.g., reflexology, acupressure); and mind-body therapies (e.g., yoga, meditation, hypnosis and guided imagery), provided on their own or as part of a multi-modality treatment regimen. The Society for Integrative Oncology (SIO), in collaboration and with the endorsement of the American Society for Clinical Oncology (ASCO), has published a number of clinical practice guidelines for the use of CIM in the treatment of symptoms among patients surviving breast cancer [15,16]; for the treatment of cancer-related pain [17]; and for anxiety and depression [18]. A number of other systematic reviews examining the effectiveness of CIM in the treatment of C-RP have since been published, though they have not yet been included in clinical practice guidelines.

The present review will examine the evidence on the use of CIM practices for the treatment of C-RP and pain-related conditions, based on the 2022 SIO-ASCO guideline [17], as well as the findings of more recently-published research and systematic reviews. Modalities such as acupuncture, massage, touch and mind-body therapies, as well as the use of herbal and other dietary supplements will be explored for this purpose. The C-RP symptom groups to be examined include general pain and symptoms related to the cancer and its invasion of adjacent structures; peri-operative and procedure-related pain; CIPN and AIA; and the treatment of anxiety-related symptoms believed to exacerbate C-RP and related symptoms. The analgesic mechanism of the CIM modalities presented will be examined as well, as will the challenges facing researchers conducting clinical research in the IO setting. Finally, the challenges facing the integration of CIM practices within the conventional oncology and palliative care setting will be addressed, with a proposal provided for the advancement of IO services across the globe.

## 2. Acupuncture

Acupuncture is an ancient Eastern medicine-based practice, in which ultra-thin (0.15–0.13 mm) needles are inserted into the epidermis and dermis at selected points, based on the principles of traditional Chinese medicine (TCM). In Western medicine, acupuncture is considered as a therapeutic option adapted from TCM using current knowledge of anatomy, physiology and pathology, and in accordance with the principles of evidence-based medicine [19]. The TCM paradigm of care has a language which differs greatly from that of current Western medical practice, with the stated goal of promoting health by harmonizing the body’s “Chi”, or energy, which flows along channels called “Meridians”; correcting imbalances in the “Yin” and “Yang” aspects of the body; and other aspects of health and disease which are beyond the scope of this paper. Acupuncture points and “meridians” can also be activated through the local application of pressure (acupressure) or by heat produced by mugwort cigars placed over the point, a technique called “Moxibustion”. The effectiveness of acupuncture and other methods activating acupuncture points is, according to TCM, assessed by evaluating the patient’s response to the treatment, as well as the quality of their radial pulse and appearance of the tongue and its layer of coating, which are believed to reflect the balance of “Chi” in the body’s “internal organs”. In today’s acupuncture practice, needles can be attached to electrodes from a 9-volt battery (“electroacupuncture”), for what is believed to be an enhanced effect.

For more than half a century scientific research has been trying to better understand the physiological effects of acupuncture, including its analgesic effects in both animal and human models. Locally, the insertion of acupuncture needles results in changes in blood flow and cellular remodeling, with reduced mechanical stress in regional mechanical receptors in a process termed “needle wrapping” [20]. A chain reaction is thus triggered, with the induction of A-beta, A-delta and C afferent fibers and release of noradrenaline, dopamine and adenosine [21]. Following needle insertion, acupuncture induces mast cell degranulation as well, releasing the mediator histamine which has significant analgesic effects, especially for neuropathic pain [22], with ligands of histamine receptors shown to modulate the analgesic action of opioids [23]. Increased mast cell density was shown to be most significant (by 55%) at TCM-identified acupuncture points [24]. Central effects following the insertion of acupuncture needles include release of endorphin, an effect inhibited in rats by the opioid blocker naloxone [25]; as well as serotonin, substance P and enkephalin [26,27,28]. In functional MRI studies in human patients, acupuncture has been shown to alter the activity and connectivity associated with pain modulation in areas of the brain comprising the “pain cortex”, which include the insula and limbic areas, as well as the somatosensory areas S1 and S2 [29,30].

Acupuncture is considered to be safe when administered by qualified and experienced practitioners, and when conducted under medical supervision [31]. At the same time, acupuncturists need to take care when treating oncology patients, especially those who are neutropenic and/or thrombocytopenic. However, it is unclear as to the absolute number of platelets or neutrophils below which acupuncture may not be administered, with many requiring a platelet count of ≥50,000/µL and neutrophil count of ≥1000/µL for this modality [32]. At the same time, acupuncture has been found to be safe in adult patients with platelet counts of <25,000 µL and neutrophil counts < 500/µL [33]; and in chemotherapy-treated pediatric patients with platelet counts <50,000/µL [33]. The risk for bleeding and post-treatment hematoma is considered to be influenced by factors such as the type of needle being used and the experience of the acupuncturist [34,35]. At the same time, risk-benefit considerations need to take into account the ability of acupuncture and other CIM modalities to significantly relieve treatment-related symptoms, with implications regarding the ability of patients to adhere to the conventional cancer treatment regimen [35].

While a large body of non-randomized and observational pragmatic studies support the effectiveness of acupuncture as a stand-alone treatment for C-RP, the number of explanatory randomized controlled trials (RCTs) proving the efficacy of this modality is extremely limited. In the 2022 SIO-ASCO guideline, acupuncture was recommended as a treatment for general or musculoskeletal pain attributed to cancer-related factors (intermediate quality of evidence; moderate strength of recommendation) [17]. In the Personalized Electroacupuncture vs. Auricular Acupuncture Comparative Effectiveness (PEACE) trial, the only large RCT in the systematic review of the guideline (*n* = 360), cancer survivors reporting musculoskeletal pain (for ≥3 months) were randomly allocated to 12 weeks of electro- or auricular acupuncture, or usual care (analgesic medications, physical therapy, and glucocorticoid injections). Electro-acupuncture was shown to reduce the severity of pain by 1.9 points on a scale of 0–10 on the Brief Pain Inventory (BPI) tool (97.5% CI, 1.4–2.4 points; *p* < 0.001), with ear (auricular) acupuncture reducing pain by 1.6 points, when compared with usual care (97.5% CI, 1.0–2.1 points; *p* < 0.001), and with both modalities showing similar effectiveness. The analgesic effects of acupuncture were found to continue for a period of up to six months post-treatment [36]. In a follow-up subgroup analysis of the PEACE trial, examining 165 breast cancer survivors, electroacupuncture was shown to reduce pain severity scores significantly more than auricular acupuncture (−0.90 (−1.45, −0.36), *p*  =  0.001) [37]. Nevertheless, the 2022 guideline gave a low score for quality of evidence and strength of recommendation for the use of acupuncture for post-operative pain, finding a significant reduction in pain in only 3 of the 9 RCTs reviewed [17].

In a multicentered, prospective RCT also not included in the SIO-ASCO guideline, intraoperative acupuncture in gynecological oncology patients was associated with significant improvement in hemodynamic and anesthesia-related parameters, as measured by decreased mean arterial pressure (MAP, *p* = 0.026) and heart rate, as well as a significant decrease in bispectral index (BIS) when compared with controls or patients undergoing touch and relaxation treatments preoperatively (*p* = 0.024). While the findings suggest an intraoperative nociceptive effect of the acupuncture treatment, further research with RCTs is needed to confirm and better understand the implications of these findings [38].

The effects of acupuncture on specific pain-related symptoms resulting from oncology treatments have been examined as well. Aromatase inhibitor-related arthralgia is a common, often debilitating adverse effect of this group of drugs, which patients with hormone-positive breast cancer are frequently required to take for at least 5 years into survivorship [39]. The 2022 SIO-ASCO guideline gave an intermediate score for level of evidence and moderate strength of recommendation for the use of acupuncture for AIA in this patient group [17]. Another common and often debilitating group of symptoms reported by oncology patients is chemotherapy-induced peripheral neuropathy (CIPN). Patients undergoing treatment with taxane drugs (e.g., paclitaxel and docetaxel), platinum-based and other drug groups frequently suffer from symptoms caused by the damage these drugs cause to peripheral nerves, which in many cases is irreversible and can lead to long-term disability. Sensory pain-related symptoms include pain, tingling and numbness, with a “pins-and-needles” sensation, as well as a loss of sensation and a wide range of motor and autonomic symptoms, all of which can further impair the patient’s QoL and function [40]. However, in the 2022 SIO-ASCO guideline, the quality of evidence for the use of acupuncture in treating CIPN was low, and strength of recommendation weak. At the same time, in a multi-centered randomized and controlled study not included in the guideline, acupuncture alone or with additional integrative oncology modalities was shown to reduce taxane-induced peripheral neuropathy-related symptoms in patients with gynecological and breast cancer, as well as providing relief for CIPN-related symptoms during oncology treatment when compared with controls receiving standard treatment (*p* = 0.038). This was most pronounced for hand numbness and tingling (*p* = 0.045); and for discomfort (*p* < 0.0001) and pain (*p* = 0.017) [41]. The methodology of the study was limited here as well, requiring further research with RCTs to confirm and better understand the implications of these findings.

In addition to the research comparing acupuncture to conventional therapies for the treatment of CRP-related symptoms, this modality has also been studied as an “add-on” treatment to conventional supportive and palliative care. In a systematic review of 15 randomized trials, it was shown that while not as effective as conventional analgesic drugs for the relief of pain, the addition of acupuncture to these drugs provided greater relief than with the drug therapy alone. However, the review concluded that the low number of RCTs included in the analysis, as well as their low methodological quality, precluded drawing firm conclusions from the findings [42]. In an RCT conducted in 104 patients with multiple myeloma with CIPN caused by the anti-cancer agent bortezomib, the addition of acupuncture to the vitamin B12 co-enzyme methylcobalamin showed that while both modalities reduced symptoms, a significantly higher effect was observed in the acupuncture-added treatment group (*p* < 0.01) [43]. Finally, acupuncture and other CIM modalities were found to reduce the need for non-opioid analgesics among patients with breast or gynecological cancer (*p* = 0.001) [44]. Here too, further research is needed to support these findings.

## 3. Massage

Massage therapy is used in Western medicine for many medical conditions, with the manipulation of soft tissue throughout the body, most commonly using Swedish or classical massage techniques [45]. Massage therapies often include stroking, kneading, applying friction and stretching muscles, promoting relaxation and addressing muscle stiffness and pain [15]. It has been shown that massage can lead to increased central release of serotonin and dopamine, while decreasing levels of the stress hormone cortisol [46]. In general, massage is considered to be safe for the oncology patient population [47], though caution is needed when treating patients with metastatic disease or with tumors located in muscles or bones [48]. The 2022 SIO-ASCO guideline recommends massage therapy for the treatment of pain in oncology patients undergoing palliative and hospice care, specifically for patients with breast cancer who develop chronic pain (low quality of evidence; moderate strength of recommendation), as well as for patients experiencing pain during palliative and hospice care (intermediate quality of evidence; moderate strength of recommendation) [17].

The evidence supporting the analgesic effects of massage for reducing patient-reported levels of C-RP requires that the practitioner-patient interaction be taken into account. Conventional analgesic drugs address only the bio-physical realm of pain, without taking into consideration related psychological, social, cultural, and spiritual aspects of suffering. During acupuncture treatments, patients are often left alone until the acupuncturist returns to either manipulate the inserted needles or to remove them. The therapeutic interaction taking place during massage is ongoing throughout the session, and is frequently accompanied by relaxing music and pleasant smells of burning essence and essential oils which may further enhance the non-specific effect of the treatment session. The association between pain and anxiety levels has been shown to be significant for both acute and chronic pain [49,50], with studies examining both situations supporting this relationship [51,52]. In an RCT examining 380 patients with advanced cancer, a series of six 30-min massage and simple-touch sessions over a two-week period was shown to both reduce pain and improve mood (*p* < 0.001). In this study, massage was shown to be superior to simple touch for immediate pain and depressed mood, with an equal benefit for sustained pain, quality of life, and use of analgesic drugs [53]. In the multicentered pragmatic clinical IMPACT trial, 298 patients with advanced cancer, suffering from moderate-to-severe pain, were treated with weekly acupuncture or massage for 10 weeks, with monthly booster sessions for up to 26 weeks. An intention-to-treat analysis using linear mixed models showed that while acupuncture significantly reduced BPI worst pain scores, with a mean change of −2.53 points (range, 0–10; 95% CI, −2.92 to −2.15), massage was also effective, with a mean change of −3.01 points (95% CI, −3.38 to −2.63). No significant difference was found between the two interventions (−0.48; 95% CI, −0.98 to 0.03; *p* = 0.07) [54]. As with the other modalities, further research is needed to support these findings.

## 4. Touch Therapies

The term “touch therapies” is used to describe a wide range of CIM modalities, some of which are based on TCM and other Eastern paradigms, with others being Western-based. Eastern and TCM modalities such as acupressure, Shiatsu, Tuina and Reiki, seek to “harmonize” the body’s “Chi” through touch. Other modalities, such as reflexology, a practice entailing the application of varying amounts of pressure to specific points on the feet or hands [55], and therapeutic touch, are focused on reducing stress and relaxing tensed muscles and soft tissues, sometimes using points believed to correlate with the body’s internal organs. As with massage, touch therapies are also considered to be safe for the oncology patient population, though caution is needed when treating patients with metastatic disease or with tumors located in muscles or bones. The 2022 SIO-ASCO guideline found that reflexology or acupressure may be offered to patients experiencing pain during systemic therapy for cancer treatment (intermediate evidence quality; moderate strength of recommendation). However, touch therapies were found to be less effective for peri-operative and procedural pain (e.g., bone marrow biopsy) in the oncology setting, with low quality of evidence and a weak strength of recommendation. This was also the guideline conclusion regarding the effectiveness of reflexology and acupressure for relief of chemotherapy-induced peripheral neuropathy [17]. However, in a single-blind, repeated measures, randomized controlled study published after the SIO-ASCO guideline, 156 patients with stage III and IV cancer receiving palliative care were randomized to acupressure, Reiki or control interventions. Numeric Pain Rating Scale scores were significantly reduced in both CIM interventions (*p* < 0.001), as was the use of analgesic drugs (*p* < 0.001) [56].

## 5. Mind-Body Medicine

The term “mind-body medicine” covers a wide range of CIM modalities that include breathing and relaxation exercises; guided imagery and hypnosis; meditation and yoga; tai chi and qigong; spiritual care, and more. Mind-body medicine is similar to touch therapies in that a significant component of its ability to reduce C-RP is likely related to its ability to reduce anxiety and emotional distress, which as stated can exacerbate both acute and chronic pain [49,50]. Mind–body therapies have been shown to induce a number of physiological effects, which can be seen on dynamic imaging and electroencephalogram (EEG) readings. In a study using functional brain magnetic resonance imaging (fMRI), healthy volunteers exhibited significant activation of the cerebellar “pain cortex” with a hypnotically-induced suggestion of localized pain, to a degree similar to that of a true pain-inducing stimulus. The findings suggest a link between central hypnosis-mediated effects and those of the neural substrate of pain [57]. In a study examining the effects of mental imagery on pain, it was shown that the intensity of painful perception of stimuli was reduced when imagining a glove covering the forearm, while increasing when imagining a lesion. The behavioral changes observed correlated with pain-related potentials measured by EEG, as manifest in N2 pain-related evoked potentials when compared to baseline [58].

While the findings of clinical research have supported the effectiveness of mind-body therapies for non-cancer-related acute and chronic pain conditions [59], the research published in the oncology setting is limited. A 2022 systematic review and meta-analysis found that a series of at least eight sessions of hypnosis can have a significant moderate-to-large effect on the reduction of musculoskeletal and neuropathic pain, when compared with controls (Hedge’s g: −0.42; *p* = 0.025 after intervention, Hedge’s g: −0.37; *p* = 0.027 after short-term follow-up) and pain interference (Hedge’s g: −0.39; *p* = 0.029) [60]. In the 2022 SIO-ASCO guideline, an intermediate level of evidence and moderate strength of recommendation was given for the use of hypnosis for procedural pain in cancer treatment or diagnostic workup [17]. In contrast, a low score for the level of evidence and weak strength of recommendation were given for yoga in patients experiencing pain after treatment for breast or head and neck cancer, and for AI-related joint pain in breast cancer; for guided imagery with progressive muscle relaxation in patients experiencing general pain from cancer treatment; and for music therapy for patients experiencing surgical pain from cancer [17]. Research into these modalities is required, to address both specific and non-specific effects of the therapeutic process.

## 6. Herbal and Dietary Supplements

The use of herbal and other dietary supplements is considered to be the most popular CIM modality in countries such as the U.S. [61], many patients believing that because these products are “natural” they are therefore both effective and safe [62]. As many as half of oncology patients do not disclose or discuss their use of these products with their oncologists and other oncology healthcare professionals [63], either because they anticipate a negative or dismissive response; they do not see this practice as relevant to their conventional medical treatment; or because they are not asked directly [64,65]. While it is true that a number of conventional oncology drugs originate from the plant world [66], most supplements are being taken by patients without medical supervision and may lead to directly toxic effects, or else negatively interact with conventional oncology and other drugs, primarily through their pharmacodynamic effects. Oncology healthcare providers who are unfamiliar with the products being used by their patients may be unable to help make informed decisions on the benefits and risks of this practice.

The systematic review of the 2022 SIO-ASCO guideline included four Chinese herbal medicinal formulas for the treatment of C-RP: two orally ingested compounds, Jinlongshe and Xiao-Ai-Tong; and two externally applied compounds, Xiao Zheng Zhitong and Shuangbai San. The externally applied Xiao-Ai-Tong was examined in patients with bone cancer, and shown to reduce the proinflammatory cytokines interleukin-1β and tumor necrosis factor-α; increase the endogenous anti-inflammatory cytokine interleukin-10 in blood; and reduce pain and prevent adverse reactions of opioid drugs [67]. However, the guideline concluded that since the efficacy of each of these formulas was shown in only one clinical study, and in light of the variability in the quality of these trials, the evidence was insufficient to make a clinical recommendation. The guideline also included a single moderate-size, randomized, double blind, placebo-controlled trial showing a beneficial effect of omega-3 fatty acids in reducing the incidence and severity of taxane-induced peripheral neuropathy in patients with breast cancer (OR = 0.3, 0.95% CI = (0.10–0.88), *p* = 0.029) [68]. A significant increase in the polyunsaturated omega-3 fatty acids eicosapentaenoic acid (tEPA) and docosahexaenoic acid (DHA) was observed in the treated group, both of which are incorporated into the phospholipid membrane of cells, including those of the central and peripheral nervous systems; and both shown to attenuate the production of proinflammatory cytokines which induce neuropathy [69]. Finally, two studies on the dietary supplement glutamine/glutamate were included, examining their effect on the incidence and severity of CIPN: the first an RCT of patients with colon cancer receiving the neurotoxic chemotherapy agent oxaliplatin, with no reduced incidence of neuropathy [70]; the second, an RCT of female patients with ovarian cancer undergoing treatment with the taxane drug paclitaxel, also with no reduction in the incidence of neuropathy but with a lower severity of pain sensation (*p* = 0.011) [71]. The conclusion of the guideline was that “no clinical recommendations can be made on the basis of these results because of low study quality and/or small sample size”. This conclusion was most likely due to the small size of the pilot studies, as well as the controversial use of nerve conduction studies for objectively assessing peripheral neuropathy [17].

Other systematic reviews have shown a beneficial effect of herbal medicinal products and formulas for the treatment of C-RP, either alone or in conjunction with conventional opioid and non-opioid analgesic mediations. A meta-analysis examining the use of East Asian herbal medicinal remedies, in combination with conventional drugs, showed significantly better outcomes with respect to response rates (risk ratio: 1.06; 95% CI: 1.04 to 1.09, *p* < 0.0001); continuous pain intensity (standardized mean difference: −1.74; 95% CI: −2.17 to −1.30, *p* < 0.0001); total duration of pain relief (standardized mean difference: 0.96, 95% CI: 0.69 to 1.22, *p* < 0.0001); performance status (weighted mean difference: 10.71; 95% CI: 4.89 to 16.53, *p* = 0.0003); and use of opioid drugs (weighted mean difference: −20.66 mg/day; 95% CI: −30.22 to −11.10, *p* < 0.0001). The herbal remedies were associated with a lower incidence of adverse events when compared to the use of conventional medicine alone [72]. In a systematic review on the TCM compound Kushen, a beneficial effect for CR-P was found as well. Kushen injections contain ethanol and water extracts from of the roots of *Sophora flavescens* Ait. (Kushen) and the tuber of *Heterosmilax yunnanensis* Gagnep. (Baituling). Clinical research on Kushen injections, not included in the SIO-ASCO guideline, found a significant improvement in physical functional status, with the relief of symptoms including pain (*p* < 0.001) [73]. The main components of Kushen injections are matrine and oxymatrine, which have been shown to regulate a wide range of inflammatory factors by blocking the TRPV1 signal transduction pathway [74]. A systematic review comprised of seven RCTs, including 521 patients with bone cancer, showed that Kushen was significantly more effective than radiotherapy or bisphosphonates for the relief of bone pain (*n* = 521, risk ratio (RR) = 1.25, 95% confidence intervals, 1.13 to 1.38, *p* < 0.0001). Despite these findings, the systematic review concluded that since the studies included were of poor methodological quality and with a small sample size, additional rigorously designed RCTs are required before Kushen can be recommended as standard care for C-RP and related symptoms [75].

## 7. Multi-Modality CIM Treatment Programs

The IO setting is patient-centered, providing a patient-tailored treatment program which acknowledges the complexity and frequent multiplicity of concerns facing patients with cancer, where pain is invariably accompanied by other symptoms and concerns [76]. In most IO programs patients are treated with more than one, and often many integrative therapies for a wide spectrum of physical, emotional and spiritual concerns. It is therefore extremely difficult for researchers to examine the effectiveness of the intervention within the framework of an explanatory research methodology (i.e., randomized, controlled trials). In order to address this limitation, pragmatic research methodologies (i.e., non-randomized and observational trials) are being employed. And while high level of evidence RCTs examining the efficacy of a treatment maximize the likelihood of observing a treatment outcome, pragmatic studies assessing the effectiveness of the IO setting can account for external patient-, provider-, and system-level factors which may moderate the true effects of the intervention [77]. Pragmatic research has shown that patients undergoing chemotherapy for breast and gynecological cancer who are adherent to a multi-modality IO patient-tailored treatment program report reduced levels of pain, with a reduced need for non-opioid analgesics (*p* = 0.01) [44]. In a prospective, pragmatic study of 815 patients undergoing chemotherapy and/or palliative care, those adhering to a multi-modal, 6-week IO treatment program reported a greater reduction in the severity of their pain when compared to those who were with low adherence to the program (*p* = 0.008) [76]. Finally, pragmatic research has shown that multi-modality patient-oriented IO treatment can improve patient adherence to the planned oncology treatment regimen (measured as relative dose intensity, RDI; *p* = 0.005), possibly through the relief of C-RP and other adverse effects of the treatment [78]. Nevertheless, IO research should still ideally be conducted within the framework of an RCT, when possible.

## 8. Challenges in Researching CIM for C-RP

Current conventional treatment options for the prevention and treatment of C-RP remain limited in both their effectiveness and safety, with many patients reporting inadequate control of their symptoms. The multifactorial pathogenesis of C-RP is often not clear, especially with oncology treatment-related complications such as CIPN and AIA. Associated anxiety can further exacerbate pain-related symptoms, which in many cases persist throughout survivorship and end-of-life care, often requiring the oncologist to reduce doses or change medications to second or third-line drugs. At the same time, oncology patients often suffer from other symptoms which are addressed during the IO consultation and treatments and which may exacerbate other C-RP-related symptoms. Currently published systematic reviews and clinical guidelines have been restricted to explanatory RCTs, which include modalities such as acupuncture, massage, touch therapies, mind-body-medicine, and the use of herbal and other dietary supplements. These modalities have been found to offer relief for general, peri-operative and procedural pain, as well as for CIPN and AIA, among other symptoms. However, CIM practices provided in the IO setting are patient-centered and most often individualized, which can be better studied using pragmatic research methodologies which reflect the “real-life” impact of these treatments on C-RP and other QoL-related outcomes. At the same time, clinical practice guidelines such as those of the 2022 SIO-ASCO publication should be considered. A study of 52 oncology healthcare professionals in Israel found that while the majority were positive in their support for the C-RP-related guideline recommendations, acknowledging their clinical relevance to patient care, they also identified a number of barriers, the most significant of which was the gap existing between rigorous science and clinical practice, with high-quality explanatory (vs. pragmatic) RCTs which do not necessarily reflect real-life clinical practice [79].

**Figure 1:**
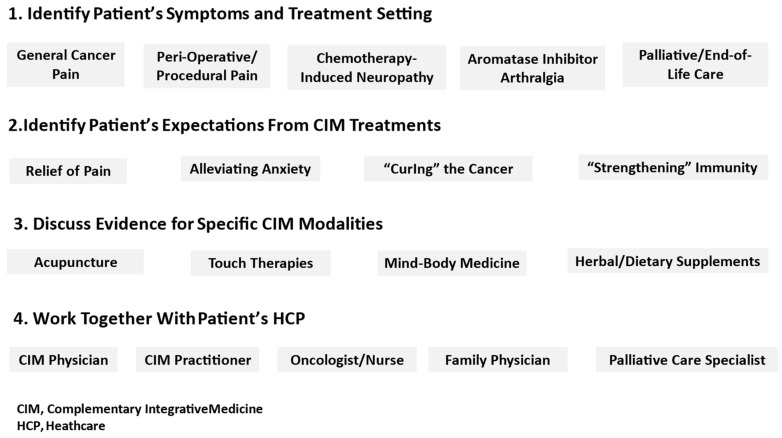
Suggested approach for implementing evidence-based CIM modality options for cancer-related pain.

A suggested approach to the implementation of the 2022 SIO-ASCO guideline, as well as the findings of other RCTs and systematic reviews, is presented in Figure 1. The IO model has much to offer in addition to conventional palliative and supportive care to oncology patients, including in the treatment of C-RP and other disabling symptoms. The collaboration between integrative and conventional healthcare providers, including the patient’s primary care physician, can ensure continuity of care in a safe and effective environment [80]. IO services are most often headed by integrative physicians, CIM-trained medical doctors who integrate these modalities in their clinical practice [81]. In addition to providing patients with evidence and guideline-based guidance on the use of CIM for C-RP and related symptoms, the integrative physician can serve as a “gatekeeper” to ensure the safety of these practices, especially with respect to the use of herbal and dietary supplements. This often entails reframing the expectations of patients and their caregivers, from “curing” their cancer or “strengthening” their immune system, to more realistic goals related to symptom relief and improved QoL [82].

## 9. Conclusions

C-RP is a prevalent and often debilitating group of symptoms associated with the patient’s cancer and its treatment. IO programs providing CIM therapies to cancer patients can potentially provide additional relief to conventional supportive and palliative care services, for C-RP and other QoL-related symptoms. The 2022 SIO and ASCO guideline, which allocate an intermediate level of evidence and a moderate strength of recommendation for a number of CIM modalities in the treatment of C-RP, should be considered. These include acupuncture for general, peri-operative/procedural and aromatase inhibitor-induced pain; reflexology or acupressure for pain during systemic therapy for cancer; and hypnosis for procedural pain, or pain due to diagnostic workup [17]. However, this paper is a narrative review and is thus non-comprehensive and presents a non-exhaustive sample of the literature, with the goal of fostering a deeper understanding of the topic [83]. Further research is therefore needed to explore the effectiveness of other CIM modalities, to be used alone or as an add-on to palliative and psycho-oncology care, within both high level of evidence explanatory RCTs and lower evidence pragmatic studies which are more reflective of the real-life IO setting.

## Data Availability

Data are contained within the article.
